# Glycerol Monolaurate, an Analogue to a Factor Secreted by *Lactobacillus*, Is Virucidal against Enveloped Viruses, Including HIV-1

**DOI:** 10.1128/mBio.00686-20

**Published:** 2020-05-05

**Authors:** Jennifer L. Welch, Jinhua Xiang, Chioma M. Okeoma, Patrick M. Schlievert, Jack T. Stapleton

**Affiliations:** a Department of Microbiology and Immunology, Carver College of Medicine, University of Iowa, Iowa City, Iowa, USA; b Medical Service, Iowa City Veterans Affairs Medical Center, Iowa City, Iowa, USA; c Department of Internal Medicine, University of Iowa, Iowa City, Iowa, USA; d Department of Pharmacology, Stony Brook University School of Medicine, Stony Brook, New York, USA; University of Rochester; University of Illinois at Chicago

**Keywords:** coronavirus, glycerol monolaurate, *Lactobacillus*, mumps virus, reutericyclin, viruses, yellow fever virus, Zika virus, human immunodeficiency virus

## Abstract

The vaginal microbiota influences sexual transmission of human immunodeficiency virus type 1 (HIV-1). Colonization of the vaginal tract is normally dominated by *Lactobacillus* species. Both *Lactobacillus* and Enterococcus faecalis may secrete reutericyclin, which inhibits the growth of a variety of pathogenic bacteria. Increasing evidence suggests a potential therapeutic role for an analogue of reutericyclin, glycerol monolaurate (GML), against microbial pathogens. Previous studies using a macaque vaginal simian immunodeficiency virus (SIV) transmission model demonstrated that GML reduces transmission and alters immune responses to infection *in vitro*. Previous studies showed that structural analogues of GML negatively impact other enveloped viruses. We sought to expand understanding of how GML inhibits HIV-1 and other enveloped viruses and show that GML restricts HIV-1 entry post-CD4 engagement at the step of coreceptor binding. Further, HIV-1 and yellow fever virus (YFV) particles were more sensitive to GML interference than particles “matured” by proteolytic processing. We show that high-pressure-liquid-chromatography (HPLC)-purified reutericyclin and reutericyclin secreted by *Lactobacillus* inhibit HIV-1. These data emphasize the importance and protective nature of the normal vaginal flora during viral infections and provide insights into the antiviral mechanism of GML during HIV-1 infection and, more broadly, to other enveloped viruses.

## Abstract (precis)

A total of 340 million sexually transmitted infections (STIs) are acquired each year. Antimicrobial agents that target multiple infectious pathogens are ideal candidates to reduce the number of newly acquired STIs. The antimicrobial and immunoregulatory properties of GML make it an excellent candidate to fit this critical need. Previous studies established the safety profile and antibacterial activity of GML against both Gram-positive and Gram-negative bacteria. GML protected against high-dose SIV infection and reduced inflammation, which can exacerbate disease, during infection. We found that GML inhibits HIV-1 and other human-pathogenic viruses (yellow fever virus, mumps virus, and Zika virus), broadening its antimicrobial range. Because GML targets diverse infectious pathogens, GML may be an effective agent against the broad range of sexually transmitted pathogens. Further, our data show that reutericyclin, a GML analog expressed by some lactobacillus species, also inhibits HIV-1 replication and thus may contribute to the protective effect of *Lactobacillus* in HIV-1 transmission.

## INTRODUCTION

Topical agents that are safe and effectively reduce transmission of multiple infectious agents are ideal candidate treatments to reduce the incidence of sexually transmitted infections (STIs). Reducing the risk of sexually transmitted infections is often centered on barrier contraceptives (1). While barrier contraceptives reduce the risk of HIV-1 sexual transmission and, presumably, that of other STIs, they are not 100% effective (2, 3). In addition, many STIs may be asymptomatic, causing significant systemic and reproductive health problems prior to diagnosis or initiation of treatment (4). Therefore, preventative approaches for STI that do not interfere with sexual norms are critically needed to curtail the estimated 340 million newly acquired STIs each year (5).

Microbicides are advantageous because of their potential activity against a range of organisms, long-term safety and effectiveness, adaptability to cultural systems, ease of use, affordability, maintenance of the normal vaginal microbiota, and tolerance at mucosal surfaces (6). Previously developed microbicides are designed to maintain the acidity of the vaginal tract and to disrupt pathogen membrane function or cellular binding (7). However, studies of existing agents have not demonstrated effectiveness, in part due to safety concerns or the risk of reporting bias (6).

Glycerol monolaurate (GML), a fatty acid formed from glycerol and lauric acid, has antimicrobial and immunoregulatory properties (8–12). Currently, GML is used as a food and cosmetic additive and is generally recognized as safe (GRAS) by the FDA. Rhesus macaque studies showed that GML was safe, and epithelial integrity was observed during long-term vaginal use (13). Further, GML was not disruptive of the normal *Lactobacillus* vaginal microbiota important to maintaining vaginal pH and did not induce inflammation (13). *In vitro* studies showed that GML reduces T cell proliferation and activation following stimulation by T cell receptor (TCR) agonists, reducing the production of TCR-induced cytokines (14).

GML has broad antibacterial activity and inhibits the growth of many Gram-positive and Gram-negative bacteria, reduces exotoxin production, and inhibits the formation of biofilms (8–10, 12). GML also inhibits growth of vaginal bacteria that increase susceptibility to HIV-1 and other STIs (9, 15). The protective properties of GML with respect to the vaginal mucosa and inhibitory effects on cytokine production suggest that GML may confer protection during HIV-1 transmission, as immune activation and inflammation increase susceptibility to HIV-1 (13). Previous *in vivo* macaque studies of simian immunodeficiency virus (SIV) vaginal transmission confirmed that GML protected subjects from acute and systemic high-dose intravaginal SIV infection (16, 17). GML also reduced HIV-1-induced secretion of proinflammatory cytokines, MIP-3α, and interleukin-8 (IL-8), further supporting the hypothesis of an immunoregulatory effect during infection (16).

Purified and human milk-derived monoglycerides provide antiviral activity against enveloped viruses, including herpes simplex virus 1 (HSV-1) and HSV-2, vesicular stomatitis virus (VSV), and visna virus, but are ineffective against nonenveloped picornaviruses, including poliovirus and rhinovirus (18–23). Previous studies showed that monoglycerides that are similar to GML inactivate enveloped RNA and DNA viruses (18–22). Although the mechanism of action is not well characterized, electron microscopy (EM) of VSV treated with linoleic acid, a polyunsaturated fatty acid, revealed disruption of the viral envelope and of particle integrity (19, 20). Similar EM results were obtained from treatment of influenza A virus and coronavirus (CoV) infections with a monolaurin mixture (22). Other studies showed that phage treated with a monoglyceride had altered sedimentation in sucrose gradient centrifugation experiments (21). These results suggest that the viral envelope may be critical for inactivation (19–21). Here, we examined how GML restricts HIV-1 and additional human-pathogenic virus infections *in vitro*. We show that GML does not interfere with CD4 receptor binding but prevents HIV-1 CXCR4 coreceptor binding. Consistent with previous studies, GML also inhibited enveloped viruses, including mumps virus, yellow fever virus (YFV), and Zika virus, but did not interfere with viral replication of nonenveloped viruses. In YFV infection, promoting envelope maturation reduces sensitivity to GML, consistent with previous findings suggesting that the viral envelope is sensitive to GML inactivation. GML is structurally related to reutericyclin, a compound secreted by *Lactobacillus*. Reutericyclin inhibits a range of bacterial pathogens (24), and *Lactobacillus-*dominated vaginal microenvironments are protective against heterosexual transmission of HIV-1. Our data suggest that reutericyclin produced by *Lactobacillus* may contribute to protection against heterosexual transmission of HIV-1 (25, 26).

## RESULTS

### GML acts on HIV-1 directly to reduce entry.

**FIG 1:**
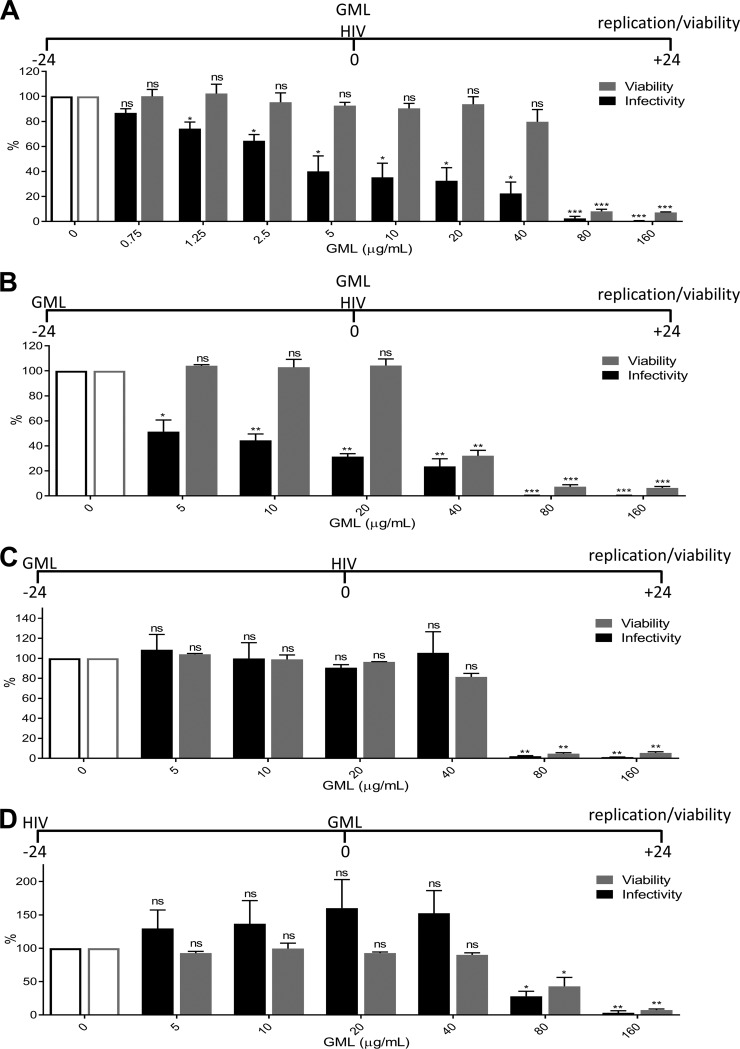
GML acts on HIV-1 directly to reduce entry. (A) Infectivity and viability after GML was added to TZM-bl cells at the time of HIV-1 inoculation. (B) Infectivity and viability after GML was added to TZM-bl cells prior to HIV-1 inoculation and maintained in GML at the time of infection. (C) Infectivity and viability after GML was added to TZM-bl cells and removed prior to HIV-1 inoculation. (D) Infectivity and viability after GML was added to TZM-bl cells postinoculation with HIV-1. Ethanol was used as a vehicle control. Vehicle-treated cells are set as the reference at 100% for infectivity and viability. TZM-bl infectivity was measured by luciferase reporter activity. Statistics data were determined by comparing infectivity or viability values from vehicle control to values from treatment. Significance was determined by Student's *t* test. *, *P* < 0.05; **, *P* < 0.01; ***, *P* < 0.001. Error bars represent SEM of results from three biological replicates each with triplicate values. ns, not significant.

GML prevented high-dose SIV vaginal infection of macaques when administered 1 h prior to challenge. Daily vaginal GML treatments were included throughout the study (16, 17). Here, we examined the kinetics of GML with respect to effects on HIV-1 replication *in vitro*. TZM-bl cells were used as these are a HeLa cell derivative engineered to express high levels of HIV-1 receptor and coreceptor (CD4 and CXCR4, respectively) and contain β-galactosidase and luciferase regulated by the HIV-1 LTR promoter to quantitate infection (27–29). Addition of GML to cells at the time of HIV-1 inoculation significantly inhibited HIV-1 replication at all concentrations of GML tested (Fig. 1A). Although high (>40 μg/ml) concentrations of GML reduced cell viability, HIV-1 was also inhibited at noncytotoxic (≤40 μg/ml) concentrations of GML (Fig. 1A). We confirmed this observation in a Jurkat CD4^+^ T cell line (see Fig. S1A to C in the supplemental material).

10.1128/mBio.00686-20.1 FIG S1 GML inhibition of HIV-1 is conserved in a CD4^+^ T cell line. (A) Viral mRNA levels, (B) p24 protein levels, and (C) viability after addition of GML to Jurkat CD4^+^ T cells at the time of HIV-1 inoculation. Ethanol was used as a vehicle control. Vehicle-treated cells were set as the reference for viral mRNA, p24, and viability data. Viral mRNA levels were measured by qRT-PCR and analyzed by relative quantification where transcripts were normalized to the GAPDH value. Viral p24 protein levels were measured by p24 ELISA. Statistics data were determined by comparing viral mRNA, p24, and viability values from vehicle control to values from treatment. Significance was determined by Student’s *t* test. *, *P* < 0.05; **, *P* < 0.01; ***, *P* < 0.001. Error bars represent SEM of results from three biological replicates. ns, not significant. Download FIG S1, TIF file, 1.2 MB. Copyright © 2020 Welch et al. 2020 Welch et al. This content is distributed under the terms of the Creative Commons Attribution 4.0 International license.

Because of the immunoregulatory profile of GML, we hypothesized that the addition of GML prior to infection might condition cells to produce an antiviral state and further enhance its protective effect. Addition of GML to cells for 24 h prior to infection, washing the cells, and incubation with HIV-1 plus GML showed similar levels of HIV-1 inhibition; however, cellular toxicity was increased by approximately 2-fold (to <40 μg/ml) by the prolonged exposure of cells to GML (Fig. 1B). Cells incubated in GML for 24 h but washed prior to HIV-1 infection did not demonstrate HIV-1 replication inhibition, and cell viability was restored to that seen in other infections with similar GML exposure times (Fig. 1C). Consistent with these findings, addition of GML 24 h after HIV-1 infection did not inhibit HIV-1 replication at noncytotoxic concentrations (≤40 μg/ml) (Fig. 1D). These data suggest that the HIV-1-inhibitory effect of GML occurs during binding and/or entry and that it does not condition cells to protect against infection.

### GML modestly reduces HIV-1 binding and more dramatically inhibits HIV-1 entry into permissive cells.

**FIG 2:**
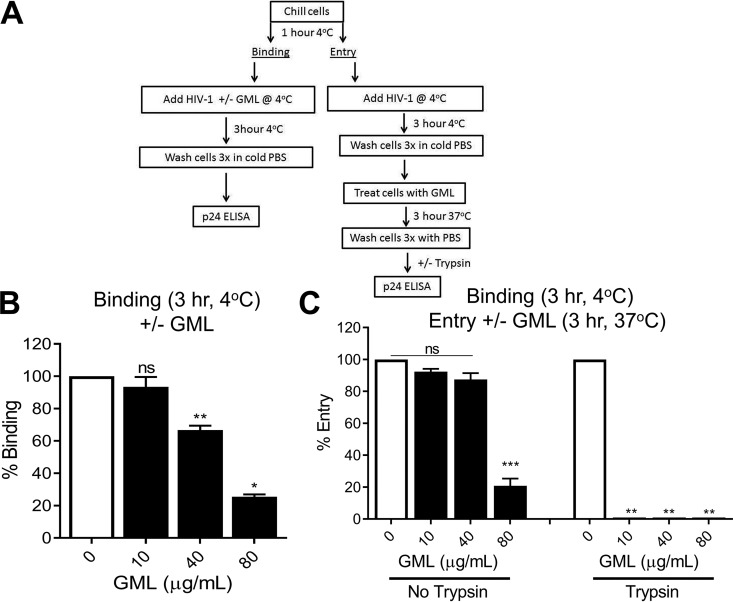
GML reduces HIV-1 binding and HIV-1 entry into permissive cells. (A) Experimental approach for quantification of HIV-1 binding and entry. (B) GML effect on HIV-1 binding to TZM-bl cells maintained at 4°C. (C) GML effect on HIV-1 entry into TZM-bl cells, without trypsin removal of surface-bound virus (left) or with trypsin removal of surface-bound virus (right). *, *P* < 0.05; **, *P* < 0.01; ***, *P* < 0.001. Error bars represent SEM of results from three biological replicates each with triplicate values. ns, not significant.

To quantify binding and entry, TZM-bl cells were maintained at 4°C for 1 h before infection. HIV-1 (NL4.3) was chilled to 4°C and applied in the presence of various concentrations of GML (Fig. 2A). Cells were incubated for 3 h at 4°C, washed in cold phosphate-buffered saline (PBS), and lysed in p24 antigen detection lysis buffer. The amount of bound HIV-1 was determined using a p24 antigen enzyme-linked immunosorbent assay (ELISA). HIV-1 cell binding was reduced by 35% using 40 μg/ml GML (Fig. 2B), which is not toxic to cells (Fig. S2A).

10.1128/mBio.00686-20.2 FIG S2 GML effects cannot be attributed to reduced cell viability. (A) Effect of GML on viability of TZM-bl cells maintained at 4°C for 3 h. Cells were warmed to 37°C for 2 h with addition of MTT reagent for assay. (B) Viability of TZM-bl cells maintained at 4°C for 3 h and warmed to 37°C for 3 h with addition of GML. (C) Viability of TZM-bl cells pretreated for 1 h with AMD3100 at 37°C with addition of GML for 24 h at 37°C. (D) Viability of HOS CXCR4^+^ CD4^−^ cells treated with soluble CD4 and GML or anti-gp120 at 37°C for 24 h. Ethanol was used as a GML vehicle control. PBS was used for sCD4 control. H_2_O was used for AMD3100 control. Statistics data were determined by comparing values from vehicle to values from treatment. Significance was determined by Student’s *t* test. Error bars represent SEM of results from three biological replicates. ns, not significant. Download FIG S2, TIF file, 1.4 MB. Copyright © 2020 Welch et al. 2020 Welch et al. This content is distributed under the terms of the Creative Commons Attribution 4.0 International license.

To assess virus entry, HIV-1 was added to cells for 3 h at 4°C, cells were washed in PBS, and GML was added (Fig. 2A). Cells were warmed to 37°C for 3 h, and total cellular p24 was measured (Fig. 2C, left). Alternatively, cell surface virus was removed by trypsin treatment following 3 h incubation at 37°C (Fig. 2C, right) and intracellular HIV-1 assessed by p24 as described above. At nontoxic GML concentrations (Fig. S2B), minimal reduction in total surface plus intracellular virus was observed compared to the no-GML control (Fig. 2C, left). However, removal of cell surface virus with trypsin completely blocked HIV-1 entry in cells treated with GML (Fig. 2C, right). These data suggest that GML partially reduced HIV-1 binding to cells at high concentrations. Thus, the overall effect of GML on HIV-1 infectivity is not likely due to interference with cellular binding. In contrast, GML completely blocked HIV-1 entry (Fig. 2C).

**FIG 3:**
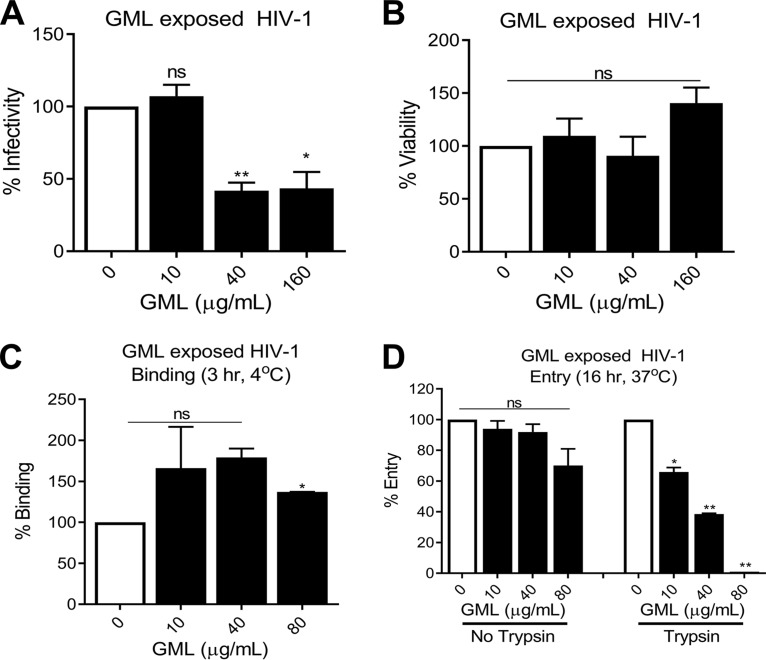
GML exposure of HIV-1 to GML impairs virus activity. GML was incubated with HIV-1 for 30 min at 37°C before removal by buffer exchange. (A and B) Infectivity (A) and cell viability (B) following addition of GML-exposed virus to TZM-bl cells. TZM-bl infectivity was measured by luciferase reporter activity. (C) Binding of GML-exposed virus added to TZM-bl cells for 3 h at 4°C. (D) The effect of GML on total cellular virus (left) and intracellular virus (right) in TZM-bl cells incubated for 16 h at 37°C. Binding and entry were quantified by p24 ELISA. For panel D, cells were treated with trypsin to proteolytically cleave surface-associated virus prior to lysis and p24 ELISA. Statistics data were determined by comparing vehicle control to treatment. Significance was determined by Student's *t* test. *, *P* < 0.05; **, *P* < 0.01. Error bars represent SEM of results from three biological replicates each with triplicate values. ns, not significant.

EM imaging of other viruses after treatment with agents similar to GML suggests that monolaurates interfere with viral replication due to changes in the viral envelope structure (19, 20, 22). To verify that the effect of GML was due to direct viral interaction, GML was added to HIV-1 for 30 min at 37°C prior to removal of GML by buffer exchange. Virus binding, entry, and infection were assessed for these GML-treated virions as described above. Replication of HIV-1 exposed to GML was significantly reduced (Fig. 3A). This did not appear to be a result of residual GML, as virus incubated in cytotoxic concentrations (160 μg/ml) of GML showed no reduction of cell viability (Fig. 3B). Reduced infectivity could not be attributed to a reduction in the ability of virus to bind to cells (Fig. 3C). However, similarly to the previous results, GML-exposed virus had a significant impairment in virus entry (Fig. 3D). Taken together, these results show that GML interactions with HIV-1 alter viral entry.

**FIG 4:**
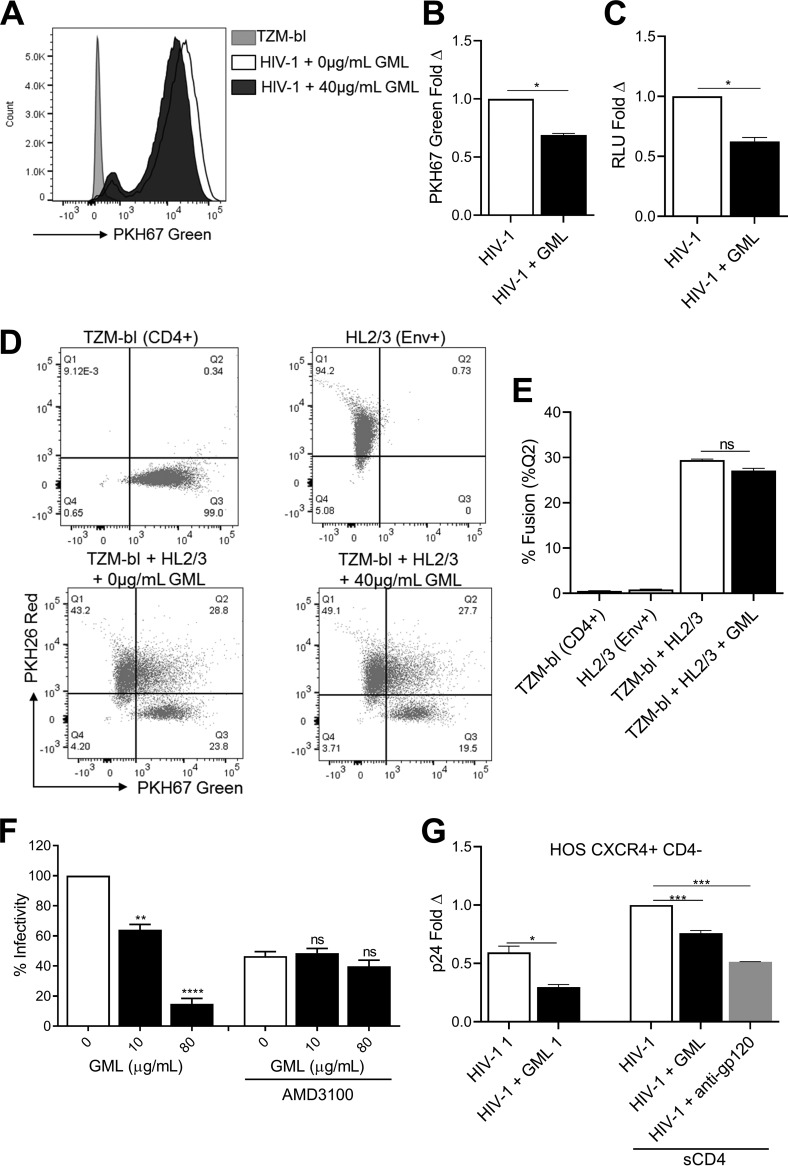
GML restricts HIV-1 binding to coreceptor. Effects of GML on binding of green fluorescence-labeled HIV-1 in TZM-bl cells were determined. (A and B) Quantification of internalized virus following trypsin removal of surface virus as counts (C) and fold data (B). (C) TZM-bl luciferase (RLU) reporter activity. Cell-cell fusion of green fluorescence-labeled TZM-bl (CD4^+^) and red fluorescence-labeled HL2/3 (Env+) in the presence of GML. (D and E) Quantification of fused cells (%Quadrant 2) double-labeled red fluorescence positive and green fluorescence positive as scatterplots (D) and fusion data (E). (F) Infectivity after treatment of TZM-bl cells with CXCR4 (AMD3100) inhibitor for 1 h prior to infection and addition of GML at the time of HIV-1 inoculation. Vehicle-treated cells in the absence of inhibitor are set as the reference at 100%. TZM-bl infectivity was measured by luciferase reporter activity. (G) p24 content after HIV-1 was treated with soluble CD4 for 2 h prior to infection and addition of GML at the time of HIV-1 inoculation of HOS CXCR4^+^ CD4^−^ cells. HIV-1 treated with soluble CD4 and vehicle control is set as the reference at a value of 1. Ethanol was used as a vehicle control. Statistics data were determined by comparing values from vehicle to values from treatment. Significance was determined by Student's *t* test. *, *P* < 0.05; **, *P* < 0.01; ***, *P* < 0.001; ****, *P* < 0.0001. Error bars represent SEM of results from three biological replicates. ns, not significant.

Addition of a noncytotoxic concentration of GML (≤40 μg/ml; Fig. 1A) to TZM-bl cells at the time of inoculation with green fluorescence-labeled HIV-1 significantly reduced intracellular HIV-1 levels (Fig. 4A and B). Cell surface virus was removed with trypsin prior to analysis. GML reduction of intracellular virus (32%) was comparable to the reduction in luciferase mediated by the HIV-1 LTR promoter in infected cells treated with GML (38%) (Fig. 4A to C). Although HIV-1 entry can be mediated via the endocytic pathway, this route is generally nonproductive (30), and productive entry occurs following binding of the gp120 trimer to its receptor (CD4) followed by conformational changes allowing binding to one of two main coreceptors and followed by fusion with the cell plasma membrane (31). These data suggest that GML restricts HIV-1 replication at a step after receptor binding (Fig. 2).

Red fluorescence-labeled TMZ-bl cells that express high levels of CD4 were coincubated with green fluorescence-labeled HL2/3 cells that express HIV-1 gp120. GML treatment of coincubated cells did not significantly reduce cell-cell fusion as measured by the percentages of cells positive for both fluorescent markers (Fig. 4D and E). Thus, GML does not interfere with HIV-1 gp120 binding to CD4, and GML does not block postbinding fusion. To determine if GML interfered with HIV-1-coreceptor interactions, TZM-bl cells were treated with a CXCR4 inhibitor (AMD3100) 1 h prior to infection, and GML was added at the time of HIV-1 inoculation. Treatment with nontoxic concentrations of GML (≤40 μg/ml; Fig. S2C) did not increase infectivity inhibition compared to effects seen with the inhibitor alone (Fig. 4F). The finding that GML did not interfere with CD4 binding (Fig. 2) and yet did not influence coreceptor blockade or fusion suggests that GML alters conformational changes in the HIV gp120 trimer structure required for coreceptor binding (Fig. 4F) (32). To evaluate if GML alters virion binding to the coreceptor, HIV-1 was incubated with soluble CD4 (sCD4) for 2 h at 37°C to induce the conformational changes required for coreceptor binding (33, 34). Addition of nontoxic GML (40 μg/ml; Fig. S2D) to sCD4-treated HIV-1 and spinoculation onto HOS CXCR4^+^ CD4^−^ cells revealed that GML significantly reduced entry of HIV-1 in CD4-negative cells (Fig. 4G). Together, these results demonstrate that GML alters HIV-1 replication by restricting viral entry after CD4 binding but before CXCR4 interactions (Fig. 4).

### GML offers broad-spectrum protection against enveloped viruses.

**FIG 5:**
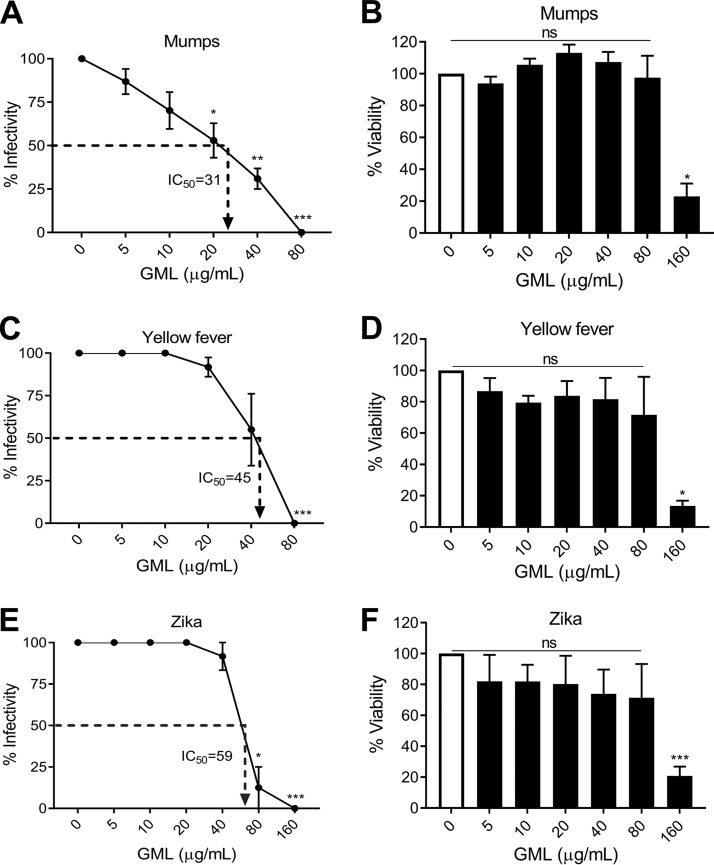
GML inhibits a broad-spectrum of enveloped viruses. GML effects on mump virus replication (A) and Vero cell toxicity (B), yellow fever virus replication (C), and cell toxicity (D) and Zika virus replication (E) and cell viability (F). Vero cells were used for assays of all three viruses, and the MOI was 1. Replication was assessed by measuring the infectivity of virus released from cells, and the 50% inhibitory concentration (IC_50_) of GML was calculated in three independent experiments. Significance was determined by Student's *t* test. *, *P* < 0.05; **, *P* < 0.01; ***, *P* < 0.001. ns, not significant. Error bars represent the SEM of results from three independent infections.

The data reported above show that GML targets HIV-1 particles by interfering with early viral life cycle events, consistent with previous studies demonstrating that other enveloped viruses are sensitive to compounds structurally similar to GML (18–22). Since virus binding and entry are required for all viral infections, we examined the effect of GML on additional viruses (35). We assessed the effect of GML on positive-strand and negative-strand RNA virus replication in permissive cell lines (mumps virus, yellow fever virus, and Zika virus). GML protected cells against infection by all three viruses at noncytotoxic concentrations (Fig. 5). Calculation of the half-maximal inhibitory concentration (IC_50_) of GML against mumps virus, yellow fever virus, and Zika virus gave values of 31, 45, and 59 μg/ml, respectively (Fig. 5). Thus, GML is not specific to HIV-1 and potentially functions as a broad-spectrum antiviral in addition to its known antibacterial and immunoregulatory properties (8–10, 12, 13, 16, 17).

**FIG 6:**
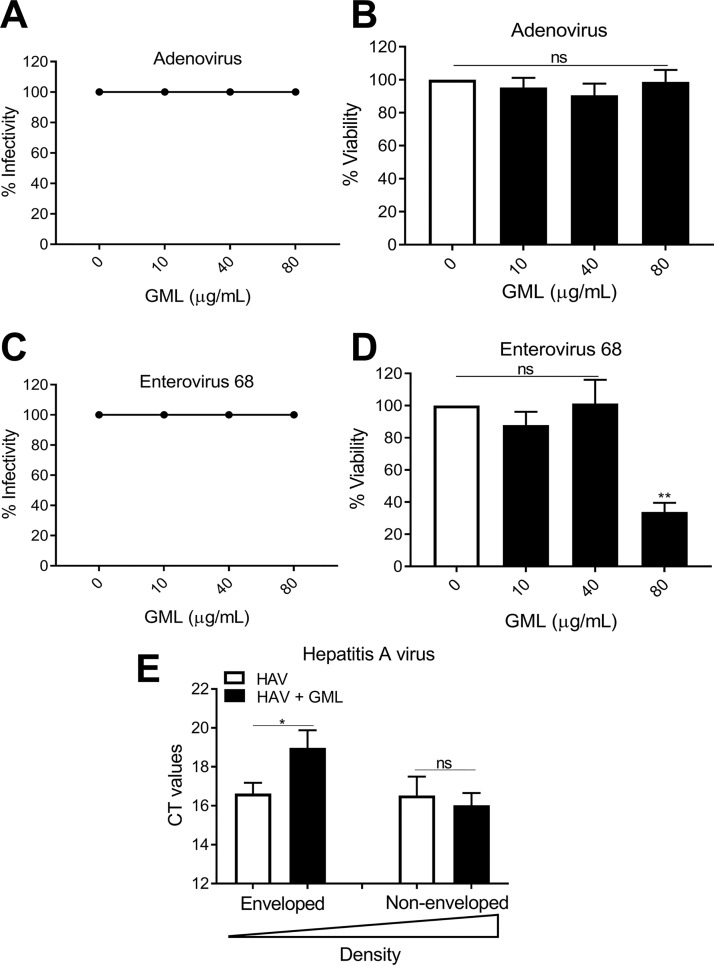
GML is inactive against nonenveloped viruses. (A to D) GML effects on adenovirus replication (A) and HEK 293 cell toxicity (B), enterovirus 68 replication (C), and MRC-5 cell toxicity (D). Vero cells were used for assays of all three viruses, and the MOI was 1 (MOI = 1). Hepatitis A virus (HAV) was separated into enveloped (eHAV) and nonenveloped (HAV) particles by isopycnic centrifugation and used to infect BSC-1 cells (MOI = 0.5). (E) GML effects on HAV RNA released following 14 days of infection. Statistics data were determined by comparing vehicle control to treatment. Significance was determined by Student's *t* test. *, *P* < 0.05; **, *P* < 0.01; ***, *P* < 0.001. ns, not significant. Error bars represent the SEM of results from three independent infections.

We also examined two positive-strand, nonenveloped RNA viruses (enterovirus 68 [EV68] and hepatitis A virus [HAV]) and a nonenveloped DNA virus (adenovirus [AdV]). GML was not inhibitory against either EV68 or AdV (Fig. 6A to D). HAV particles may exist as either nonenveloped (dense) or enveloped (light) viral particles (36, 37). Following isopycnic banding in cesium chloride gradients (38), particles were separated into purified light (enveloped) and heavy (nonenveloped) HAV particles. GML inhibited replication of light HAV particles, whereas GML had no effect on the heavy particles (Fig. 6E). These data and similar findings by others further indicate that monoglycerides like GML interfere with viral replication through direct effects on the viral envelope.

### Envelope maturation mediates viral sensitivity to GML.

**FIG 7:**
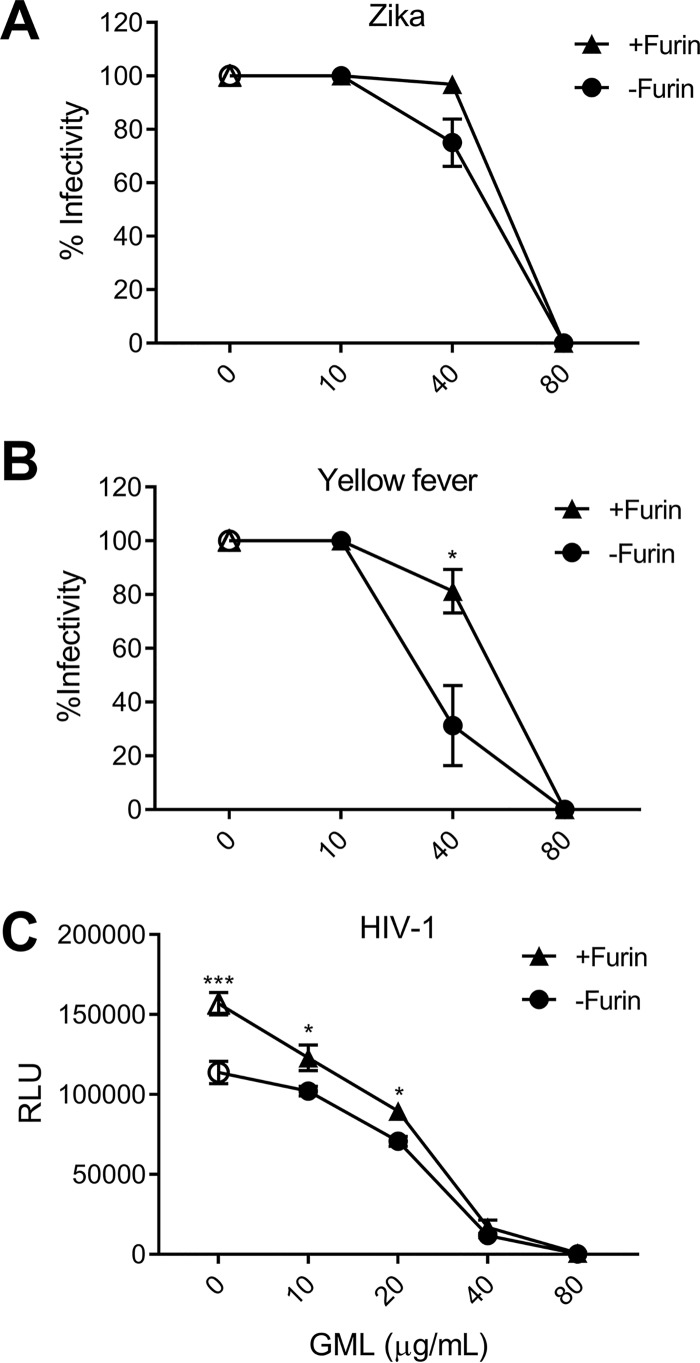
Envelope maturation influences the antiviral effect of GML. Viruses sensitive to furin-mediated envelope maturation were incubated with 50 U recombinant furin (+Furin) or medium control (-Furin) for 2 h at 37°C and pH 5.8. +Furin and -Furin viruses were treated with GML. GML effects on Zika virus infection (with or without furin) (A), yellow fever virus (with or without furin) (B), and HIV-1 (with or without furin) (C) are indicated. YFV and Zika virus were studied in Vero cells (MOI 1.0), HIV-1 was evaluated using TZM-bl cells, and infectivity was measured by luciferase reporter activity. Statistics data were determined by comparing +Furin to –Furin for each treatment concentration. Significance was determined by Student's *t* test. *, *P* < 0.05; **, *P* < 0.01; ***, *P* < 0.001. Error bars represent SEM of results from four biological replicates.

The inhibitory concentrations differed for mumps virus, yellow fever virus, and Zika viruses. Many enveloped viruses require processing of their envelope glycoproteins to mediate entry (39). For flaviviruses, a cellular protease (furin) is responsible for proteolytic cleavage of the precursor envelope (prM) proteins. The uncleaved prM conformation prevents viral fusion (40). Although furin cleavage results in “maturation” of virus particles, virus populations consist of a mixture of particles with various degrees of maturation and furin cleavage is not required for all functions of viral envelope proteins (39, 41). Viruses with a higher proportion of cleaved prM have higher specific infectivity (42). We hypothesized that GML interaction may be enhanced in “immature” flaviviruses on the basis of increased access of GML to the viral envelope and that, by increasing the proportion of mature particles with furin treatment, flaviviruses are rendered more sensitive to GML inhibition. Zika virus was less sensitive to GML than YFV, and furin treatment did not change the GML sensitivity of Zika virus (Fig. 7A). In contrast, incubation of yellow fever virus in furin increased the IC_50_ by 10 μg/ml (Fig. 7B). Similarly, HIV-1 infectivity was increased following incubation in furin, although this increase was more modest (Fig. 7C). The reduced sensitivity to GML following furin treatment for yellow fever virus and HIV-1 suggests that the maturation state of viral particles is important in GML antiviral activity and that Zika virus prM cleavage was more efficient than YFV cleavage in our production cell lines. The efficiency of Zika virus prM cleavage is currently unknown (43). Unfortunately, monoclonal antibodies that distinguish mature and immature Zika and YFV particles have not been described, and the available polyclonal antibodies do not distinguish the size differences of PrM and M. Thus, though the data are consistent with the hypothesis that GML access lipids in the YFV immature particles, further studies are needed for confirmation.

### An analogue to GML secreted by *Lactobacillus* inhibits HIV-1 infection.

**FIG 8:**
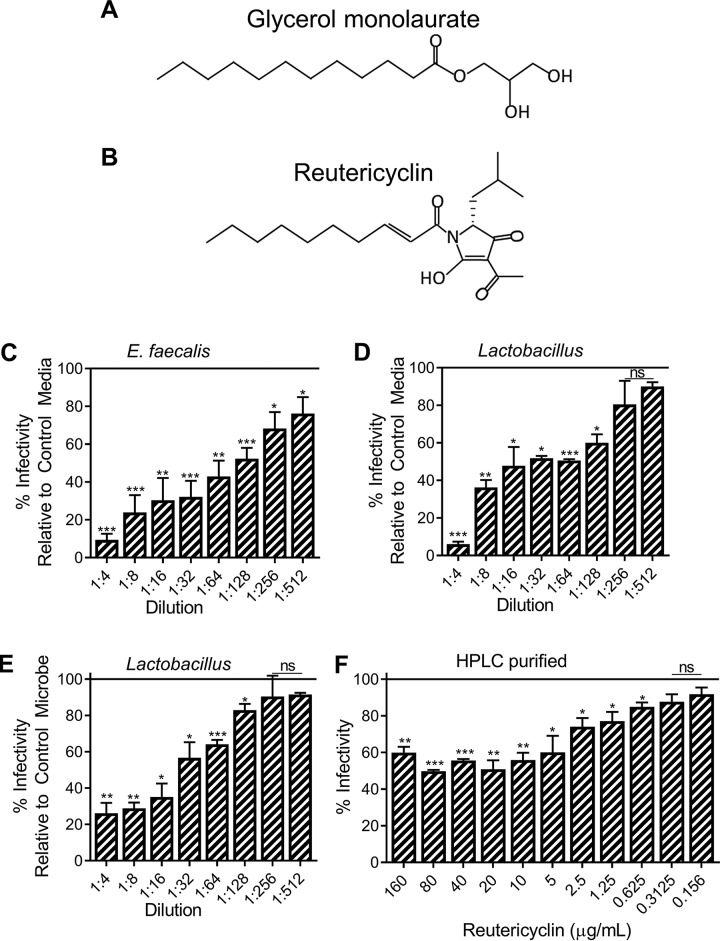
A GML analog (reutericyclin) secreted by *Lactobacillus* inhibits HIV-1 infection. (A and B) Molecular structures of (A) glycerol monolaurate (GML) and (B) reutericyclin. The effect of Enterococcus faecalis-conditioned and Lactobacillus reuteri-conditioned media (reutericyclin containing) or Lactobacillus plantarum-conditioned medium (nonreutericyclin control) on HIV-1 infection was assessed. (C and D) HIV-1 infectivity relative in the presence of E. faecalis-conditioned (C) or L. reuteri-conditioned (D) media relative to control media. (E) Similarly, HIV-1 infectivity in L. reuteri-conditioned media relative to L. plantarum media is shown. (F) Effect of HPLC-purified reutericyclin on HIV-1 infectivity. DMSO served as the vehicle control. Statistics data were determined by comparing vehicle control to treatment. Significance was determined by Student's *t* test. *, *P* < 0.05; **, *P* < 0.01; ***, *P* < 0.001. Error bars represent SEM of results from three biological replicates. ns, not significant.

Reutericyclin, a GML analogue, is secreted by isolates of Lactobacillus reuteri as well as Enterococcus faecalis (Fig. 8A and B). Reutericyclin inhibits a broad spectrum of bacterial organisms, but its inhibitory profile is unreported for viruses (24). Nevertheless, previous studies found that Lactobacillus reuteri enhances host resistance to viral infections, possibly through regulation of the microenvironment and through the secretion of antiviral metabolites (44). Incubation of HIV-1 with various dilutions of concentrated reutericyclin-containing media produced by E. faecalis and Lactobacillus reuteri significantly reduced infection at even highly diluted concentrations (Fig. 8C to E). Media without the microbe or conditioned media in the presence of a non-reutericyclin-producing strain of Lactobacillus plantarum served as controls (Fig. 8C to E). Media without bacteria and conditioned media from non-reutericyclin-producing Lactobacillus plantarum were noncytotoxic and also reduced infection, though conditioned media from reutericyclin-producing microbes inhibited HIV-1 to a significantly greater extent (Fig. S3A and B). Reduced infection by our controls indicated that microbe growth media may contain inhibitory components and that *Lactobacillus* may produce other HIV-1-inhibitory metabolites. Others have reported non-reutericyclin-based mechanisms of *Lactobacillus*-mediated inhibition of HIV-1, including secretion of other antimicrobial factors (24, 25). Because reutericyclin-containing media from bacterial secretions contain additional metabolites, high-pressure-liquid-chromatography (HPLC)-purified reutericyclin was used to validate the HIV-1-inhibitory effect of reutericyclin. HPLC-purified reutericyclin was noncytotoxic (Fig. S3B) and inhibited HIV-1 infection by 40% to 50% (Fig. 8F), suggesting that reutericyclin is an effective inhibitory factor against HIV-1 and supporting the observation that *Lactobacillus*-dominated vaginal microenvironments are protective against HIV-1. We hypothesize that microbe-produced reutericyclin is more inhibitory than HPLC-purified reutericyclin due to the secretion of additional antiviral factors in the media. Others have reported antimicrobial properties of reutericin and reuterin secreted by Lactobacillus reuteri, though antiviral effects (45–47) have not been studied. These results support the hypothesis that *Lactobacillus* may secrete inhibitory factors in addition to contributing other protective functions such as maintenance of vaginal pH (48).

10.1128/mBio.00686-20.3 FIG S3 Reutericyclin and nonreutericyclin controls in HIV-1 infection. Effects of microbe growth media, Lactobacillus plantarum*-*conditioned media (nonreutericyclin), Enterococcus faecalis-conditioned and Lactobacillus reuteri-conditioned media (reutericyclin containing), and HPLC-purified reutericyclin on (A) HIV-1 infection and (B) HIV-1 viability in TZM-bl cells were assayed. Data represent infectivity and viability relative to HIV-1 in the absence of microbe controls (set at 100%). TZM-bl infectivity was measured by luciferase reporter activity analysis. Error bars represent SEM of results from three biological replicates each with triplicate values. Download FIG S3, TIF file, 1.9 MB. Copyright © 2020 Welch et al. 2020 Welch et al. This content is distributed under the terms of the Creative Commons Attribution 4.0 International license.

## DISCUSSION

Sexually transmitted infections are a significant cause of global disease, with an estimated 1 million people infected daily (49). STIs may cause health complications and increase susceptibility to secondary infections, including HIV-1 (49). Because STIs include a diverse and expansive range of pathogens, preventative strategies that incorporate microbial control agents are needed. GML has potential to be a unique microbicide, as it has an established safety profile, its antimicrobial properties include activity against bacteria and viruses, and it regulates immune responses (8–10, 12–14, 16, 17).

**FIG 9:**
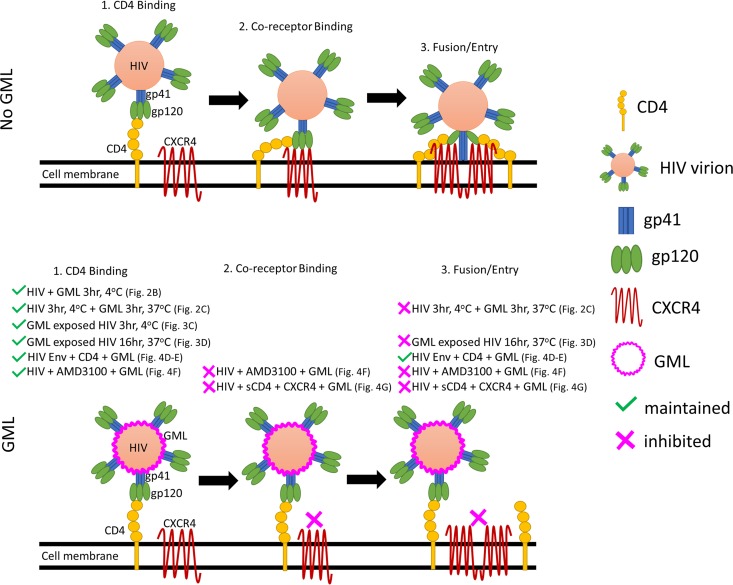
GML reduces HIV-1 entry by interference with coreceptor but not CD4 binding. In the absence of GML, HIV-1 gp120 binding to CD4 induces conformational changes that facilitate gp120 binding to the coreceptor (CXCR4). Coreceptor binding triggers exposure of gp41 and fusion of viral and cellular membranes. Our data show that HIV-1 gp120 binds CD4 in the presence of GML but that binding to the coreceptor is inhibited, resulting in reduced fusion/entry. References to the included experimental results shown in the other figures are given in parentheses.

Previous studies (18–22) and our data indicate that GML and similar monoglycerides are potent antiviral agents against diverse viruses. Our data provide new insights into the mechanism of action of GML. Incubation of permissive cells with GML or its analogue reutericyclin at the time of infection inhibited HIV-1. Although the inhibition was due partly to a reduction in virus binding to CD4^+^, the major step of the HIV-1 life cycle inhibited by GML was viral entry following CD4^+^ binding. Our data show that GML prevents virion binding to the coreceptor (Fig. 9). In addition to the antiviral mechanism of GML against virus replication, other studies found that GML also blocks immune signaling (14, 16). Thus, it is likely that GML inhibits HIV-1 and other viruses by multiple mechanisms.

GML was active against three additional enveloped viruses (yellow fever virus, mumps virus, and Zika virus) but was not active against two nonenveloped viruses (adenovirus and enterovirus 68). Separation of hepatitis A virus into nonenveloped and enveloped virions confirmed the importance of viral envelopes for GML antiviral activity. These findings are consistent with those reported by others showing that monoglycerides inhibited enveloped HSV-1 and HSV-2, VSV, and visna virus infections but were unable to restrict nonenveloped poliovirus or rhinovirus infection (18–22). We further show that GML effects on enveloped viruses are influenced by the state of envelope maturation. It remains unknown whether GML induces conformational changes to the viral lipid envelope or interferes with envelope interactions with host factors mediating virus binding, fusion, or other steps in the entry process. EM imaging and sedimentation analysis data from similar studies indicate significant conformational disruption of the viral envelope (18–22).

Relevant to the broad activity against enveloped RNA viruses, previous studies found that a monolaurin mixture disrupted the viral envelope of a coronavirus (CoV) (22). Although GML is unlikely to completely prevent CoV infection, GML or a monolaurin derivative might reduce transmission of the newly discovered coronavirus, 2019-nCoV (SARS-CoV-2), in high-risk areas such as nursing homes, cruise ships, and health care settings. An unpublished study (PMS) found that administration of a 5% GML gel nasally to 50 individuals did not produce any adverse effects. This formulation prevented transmission of high-dose SIV infection in rhesus monkeys (17). Therefore, GML is a potentially safe and effective antiviral agent against novel enveloped viruses.

GML is an analogue to *Lactobacillus*-secreted reutericyclin. The normal flora of the vaginal tract is dominated by *Lactobacillus* and protective against HIV-1. *Lactobacillus* species may prevent infection through the secretion of antiviral factors, by maintenance of an acidic vaginal pH that inactivates virions, and by disruption of virion binding to cell surface molecules (25, 50). Our data suggest that reutericyclin released by *Lactobacillus* may be a secreted factor that contributes to the protective effect induced by reutericyclin-expressing bacteria. Although reutericyclin is known to inhibit bacteria, no previous reports have shown activity against viruses (24). There are structural similarities between GML and reutericyclin, and these may provide insights into the inhibitory action and facilitate the development of more efficacious derivatives. The importance of fatty acid chain length and head group polarity with respect to virus inhibition is not yet known. Similar studies showed that 12-carbon lauric acid was the most effective of various carbon chain lengths at inhibiting Junin virus and vesicular stomatitis virus. However, those groups concluded that lauric acid disrupted maturation, a late viral life cycle event, rather than early life cycle events (51, 52). Others showed that HIV-1, HSV, and hepatitis C virus are inhibited by contact with medium-chain saturated and long-chain unsaturated fatty acids and 1-monoglycerides of medium-chain and long-chain fatty acids. These fatty acids and their derivatives disrupted the viral envelope, suggesting an early effect (19, 53–55). Therefore, the fatty acid chains of GML and reutericyclin may be more important than the polarity of the head group during enveloped virus inhibition.

## MATERIALS AND METHODS

### Cells.

TZM-bl (NIH AIDS Reagent Program), HEK293 (NIH AIDS Reagent Program), HL2/3 (NIH AIDS Reagent Program), HOS CXCR4^+^ CD4^−^ (NIH AIDS Reagent Program), MRC-5 (Sigma-Aldrich), BSC-1 (ATCC), and Vero (ATCC) cells were maintained in Dulbecco’s modified Eagle’s medium (DMEM) (Gibco-BRL/Life Technologies) with 5% exosome-depleted fetal bovine serum (FBS) (Gibco), 100 U/ml penicillin, 100 μg/ml streptomycin, 100 μg/ml sodium pyruvate, and 0.3 mg/ml l-glutamine (Invitrogen, Molecular Probes). HOS CXCR4^+^ CD4^−^ cells were also supplemented with 1.0 μg/ml puromycin. Jurkat E6-1 (NIH AIDS Reagent Program) cells were maintained in RPMI 1640 (Gibco-BR/Life Technologies) with the same supplements. Cell viability was assessed by 3-(4,5-dimethyl-2-thiazolyl)-2,5-diphenyl-2H-tetrazolium bromide (MTT) assay as previously described, and vehicle-treated cells represent 100% viability (56).

### Viruses.

HIV-1 NL4.3 virus was produced by Lipofectamine 2000 transfection of pNL4.3 plasmid (NIH AIDS Reagent Program) into HEK293 cells per the instructions of the manufacturer (Invitrogen) (57). HIV-1 titers were determined by EnzChek reverse transcriptase (RT) assay (Life Technologies) and luciferase reporter relative light units (RLU) were used for quantitation in analyses of infected TZM-bl cells (Steady-Glo; Promega) (58, 59). HIV-1 NL4.3 virus assays were completed with 100,000 RLU. Hepatitis A virus (HM175; ATCC), enterovirus 68 (US/IL/14-18952; ATCC), yellow fever virus (17D; Sanofi), mumps virus (Jeryl Lynn; Merck), adenovirus (type 5; University of Iowa Viral Vector Core), and Zika virus (PR; kindly provided by Wendy Maury at the University of Iowa) titers were determined in appropriate cell lines by 50% tissue culture infective dose (TCID_50_) determinations or quantitative reverse transcriptase PCR (qRT-PCR) analysis of viral RNA as previously described (60). All virus stocks were stored at –80°C.

### Glycerol monolaurate (GML).

GML (Colonial Company, South Pittsburg, TN) was solubilized in ethanol (100%) to a stock concentration of 100 mg/ml, and stock preparations were diluted to working concentrations in cell culture media. GML aggregates in cell culture media at high concentrations, but aggregation may be minimized at working concentrations under 200 μg/ml. Dilution of ethanol in cell culture media served as the vehicle control. GML stock and working concentrations were stored at room temperature.

### Isopycnic centrifugation of HAV.

HAV was layered on top of a CsCl solution and centrifuged at 100,000 × *g* for 1 week at 10°C by the use of a SW41Ti rotor (Beckman Coulter) (38). Approximately 20 fractions (0.5 ml) were collected, and HAV was quantified by one-step qRT-PCR. Density-light (enveloped) particles equilibrated near the gradient top, whereas density-heavy (nonenveloped) particles equilibrated near the bottom. CsCl was removed from virus fractions by dilution in PBS and centrifugation at 100,000 × *g* for 2 h at 10°C in a SW41Ti rotor (Beckman Coulter). Pelleted virus was resuspended in media before infection (61, 62).

### One-step qRT-PCR viral RNA.

HAV RNA was extracted from infected supernatants by the use of a QiAamp viral RNA kit (Qiagen). PCRs included 0.5 μM (each) HAV forward and reverse primer, 0.2 μM HAV probe, and 11 μl of viral RNA in a Platinum quantitative RT-PCR ThermoScript one-step system (Invitrogen). PCRs were completed using a model 7500 qRT-PCR system (ABI) under the following conditions: 50°C for 20 min, 95°C for 2 min, and 40 cycles of 95°C for 15 s and 58°C for 1 min. The HAV primers and probes used were as follows: HAV forward (F) primer (5′-GGTAGGCTACGGGTGAAAC-3′), HAV reverse (R) primer (5′-AACAACTCACCAATATCCGC-3′), and HAV probe (FAM [6-carboxyfluorescein]-5′-CTTAGGCTAATACTTCTATGAAGAGATGC-3′-TAMRA [6-carboxytetramethylrhodamine]) (IDT).

### HIV-1 binding and entry.

To quantify binding and entry, TZM-bl cells were maintained at 4°C for 1 h before addition of virus. For HIV-1 binding experiments, various concentrations of GML were coincubated with HIV-1 NL4.3 for 30 min at 4°C. Coincubated GML/virus was added to cells for 3 h at 4°C. After that step, cells were washed 3 times with cold PBS before cell lysis and evaluation of p24 by p24 ELISA (ZeptoMetrix) per the manufacturer’s instructions. For HIV-1 entry experiments, HIV-1 NL4.3 was added to cells for 3 h at 4°C. After that step, virus was removed and cells were washed 3 times with cold PBS to remove unbound virus. Cells were then treated with various concentrations of GML for 3 h at 37°C. Cells were again washed 3 times with PBS before treatment with medium control or trypsin to remove cell surface-associated virus. Cells were then lysed and evaluated for p24 content (ZeptoMetrix). The p24 content of vehicle (control)-treated cells was set at 100%. For flow cytometry analysis of HIV-1 entry, HIV-1 NL4.3 was subjected to green fluorescence labeling with a PKH67 green fluorescent cell linker kit (Sigma-Aldrich). Virus was pelleted at 100,000 × *g* for 1 h at 4°C and resuspended in 2 ml diluent C containing 4 μl PKH67 dye. Solutions of virus and dye were incubated for 5 min at room temperature. Staining was stopped by addition of an equal volume of FBS. Labeled virus was washed twice in DMEM and pelleted at 100,000 × *g* for 1 h at 4°C for each wash. After the final wash, labeled virus was resuspended in DMEM. Green fluorescence-labeled NL4.3 and 40 μg/ml GML or ethanol control were added to TZM-bl cells and incubated for 24 h at 37°C. Cells were treated with trypsin and evaluated by the use of an LSR II flow cytometer (BD Biosciences) and FlowJo software (TreeStar) or a Steady-Glo (Promega) luciferase assay. Vehicle (control)-treated cells were set at a value of 1.

### Cell-cell fusion.

HL2/3 (gp120^+^) cells were subjected to red fluorescence labeling with a PKH26 red fluorescent cell linker kit (Sigma-Aldrich) per the manufacturer’s instructions. TZM-bl (CD4^+^) cells were subjected to green fluorescence labeling with a PKH67 green fluorescent cell linker kit (Sigma-Aldrich) per the manufacturer’s instructions. Fluorescence-labeled cells were coincubated in equal cell numbers with 40 μg/ml GML or ethanol control for 24 h at 37°C. Cells were treated with trypsin and analyzed by the use of an LSR II (BD Biosciences) flow cytometer and FlowJo software (TreeStar). Fusion was determined as percent Q2 (colabeled red+/green+).

### GML-exposed HIV-1.

Various concentrations of GML were coincubated with HIV-1 NL4.3 for 30 min at 37°C before GML was removed from virus by 100K centrifugal filtering (Centriprep). GML-free virus was then used for infection of TZM-bl cells for 24 h, evaluation of HIV-1 binding as described above, and determination of HIV-1 entry as described above with the exception that virus was incubated on cells at 37°C for 16 h before washing, medium control or trypsin treatment, and evaluation of p24 content.

### Reutericyclin.

Enterococcus faecalis and Lactobacillus reuteri were cultured overnight at 37°C in Todd-Hewitt and MRS broth, respectively. Bacterial cells were removed from reutericyclin-containing supernatants by centrifugation. Four volumes of pure ethanol were added to reutericyclin-containing supernatants and incubated at room temperature overnight to precipitate molecules at levels above 15K. The ethanol-reutericyclin supernatant was centrifuged at 2,000 × *g* for 20 min to remove large molecules. The reutericyclin supernatant was concentrated by centrifugal filtering and dried under a laminar flow hood before resuspension (10×) in distilled water. Infections were completed by diluting the concentrated reutericyclin in cell culture media. Bacterial culture broth with no microbe or cultured with non-reutericyclin-producing bacteria (Lactobacillus plantarum*;* kindly provided by Laynez Ackermann at the University of Iowa) treated the same was used as a control. HPLC-purified reutericyclin (ChemFaces) was solubilized in dimethyl sulfoxide (DMSO). Working concentrations were diluted in complete cell culture media. An equivalent dilution of DMSO in cell culture media was used as a vehicle control. Secreted or HPLC-purified reutericyclin was incubated for 30 min at 37°C with 100,000 RLU HIV-1 NL4.3 before infection. Infection of TZM-bl cells was assessed after 24 h by RLU luciferase assay (Steady-Glo; Promega).

### Infectivity assays.

HIV-1 NL4.3 virus (100,000 RLU) was coincubated with various concentrations of GML for 30 min at 37°C before TZM-bl cell infection (co-HIV-1) was performed. Various concentrations of GML were added to TZM-bl cells for 24 h before removal, PBS washing, and infection with 100,000 RLU NL4.3 virus for an additional 24 h (pre-HIV-1). TZM-bl cells were infected with 100,000 RLU NL4.3 virus for 24 h followed by removal, PBS washing, and treatment with various concentrations of GML for an additional 24 h (post-HIV-1). At the indicated time points, cells were lysed and measured for luciferase reporter activity (Steady-Glo; Promega). Jurkat cell infection was completed using the same method, but the results were measured for viral RNA by qRT-PCR using primers specific to HIV-1 Gag-pol and the housekeeping gene GAPDH (glyceraldehyde-3-phosphate dehydrogenase) as previously described (57) and for p24 protein levels by p24 ELISA (ZeptoMetrix) per the manufacturer’s instructions. All other viruses were incubated with various concentrations of GML for 30 min at 37°C before cell infections were performed using the following viruses: mumps virus (Vero multiplicity of infection [MOI] = 0.1), yellow fever virus (Vero MOI = 1), Zika virus (Vero MOI = 1), adenovirus (HEK293 MOI = 1), enterovirus 68 (MRC-5 MOI = 1), and HAV (BSC-1 MOI = 0.5). GML/infectious medium was replaced 24 h postinfection with complete media containing 2% FBS. Infectivity was assessed by cytopathic effect (CPE) once a CPE was observed in 100% of infection control wells for CPE-producing viruses. HAV-infected cultures were maintained for 14 to 21 days before one-step qRT-PCR of viral RNA was performed. Viability was determined at the time of infectivity assessment, and treatments were normalized to infection with vehicle control. Data corresponding to 50% inhibitory concentration (IC_50_) doses were calculated from three independent experiments.

### Furin-treated viruses.

HIV-1 NL4.3 (100,000 RLU), Zika (MOI = 1), and yellow fever (MOI = 1) viruses were incubated with 50 U recombinant furin (New England Biolabs) or medium control for 2 h at 37°C and pH 5.8 (63). Viruses treated in the presence of furin (+Furin) or in the absence of furin (-Furin) were then diluted to reach a 3-ml volume and incubated with GML, and infections carried out as described above (pH 6.8 to 7.2).

### CXCR4 inhibitor.

An inhibitor that blocks HIV-1 binding to CXCR4 (1 μM AMD3100, Sigma-Aldrich) was added to TZM-bl cells for 1 h at 37°C before infection with HIV-1 NL4.3. GML was added at the time of HIV-1 inoculation, and cells were maintained in the inhibitor for the duration of the infection. Infection of TZM-bl cells was assessed after 24 h by RLU luciferase assay (Steady-Glo; Promega).

### CD4-independent entry.

A 10× higher concentration of HIV-1 NL4.3 than used in infectivity assays described above was incubated with a PBS control or 40 μg/ml soluble CD4 (sCD4) (R&D Systems) for 2 h at 37°C. Coincubation of virus with sCD4 and 10 μg/ml anti-gp120 (17b; NIH AIDS Reagent Program) was used as a control. A 40-μg/ml volume of GML or ethanol control was added at the time of HIV-1 inoculation of HOS CXCR4^+^ CD4^−^ cells. Cells were subjected to spinoculation at 1,200 × *g* for 2 h at 30°C. Cells were then incubated for 24 h at 37°C. Cells were treated with trypsin, lysed, and evaluated for p24 content (R&D Systems).

### Statistics.

Two-tailed *t* test *P* value (GraphPad Prism) calculations determined statistical significance (*, *P* < 0.05; **, *P* < 0.01; ***, *P* < 0.001; ns, not significant). Error bars represent standard errors of the means (SEM) of results from triplicate experiments.
