# Supplementary figures and images for: Glycerol Monolaurate, an Analogue to a Factor Secreted by Lactobacillus, Is Virucidal against Enveloped Viruses, Including HIV-1
Source: mBio. 2020 May 5;11(3):e00686-20. doi: 10.1128/mBio.00686-20 (PMC7201201; doi:10.1128/mBio.00686-20)

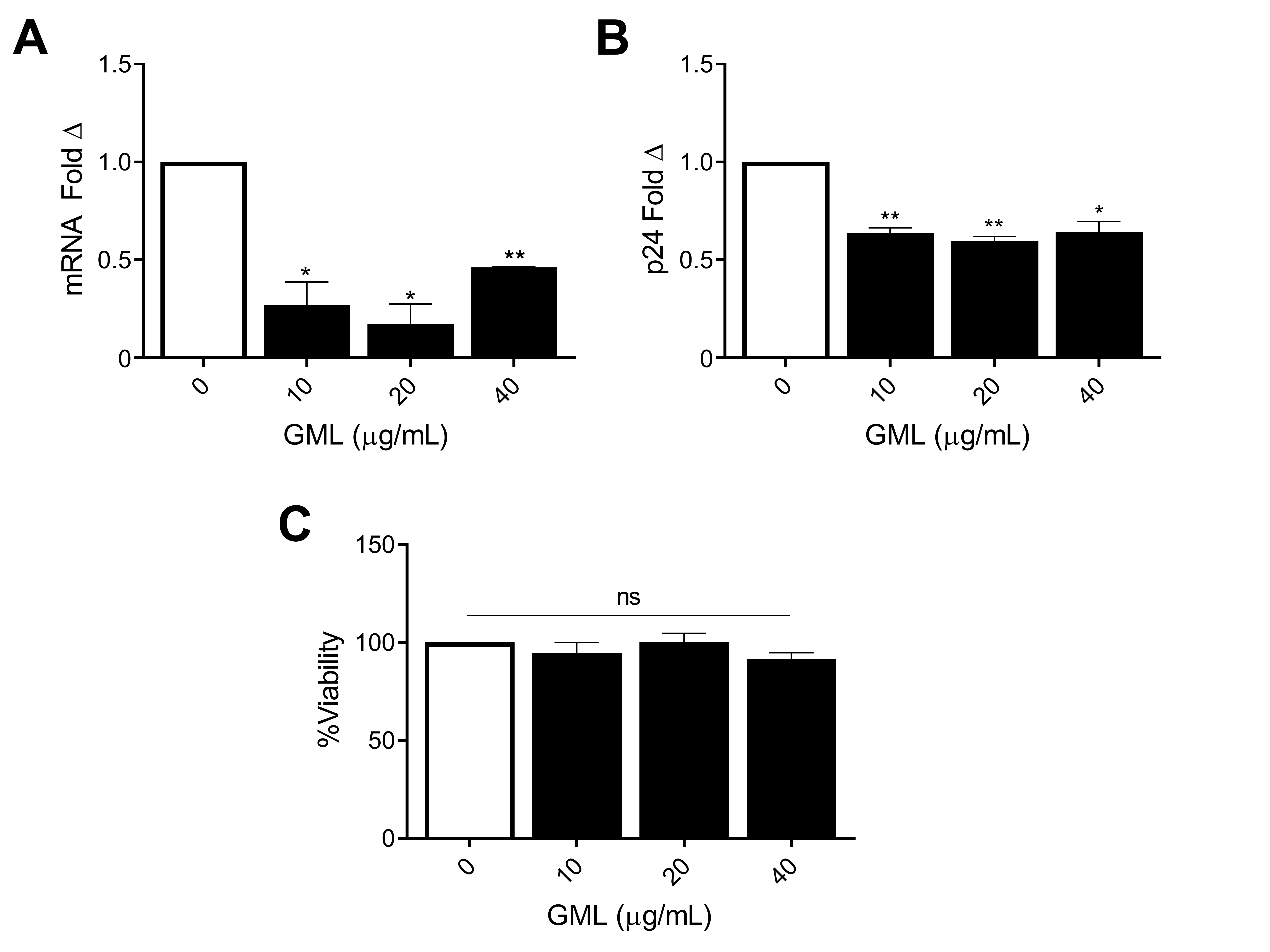

Supplement: FIG S1 [file mBio.00686-20-sf001.tif]

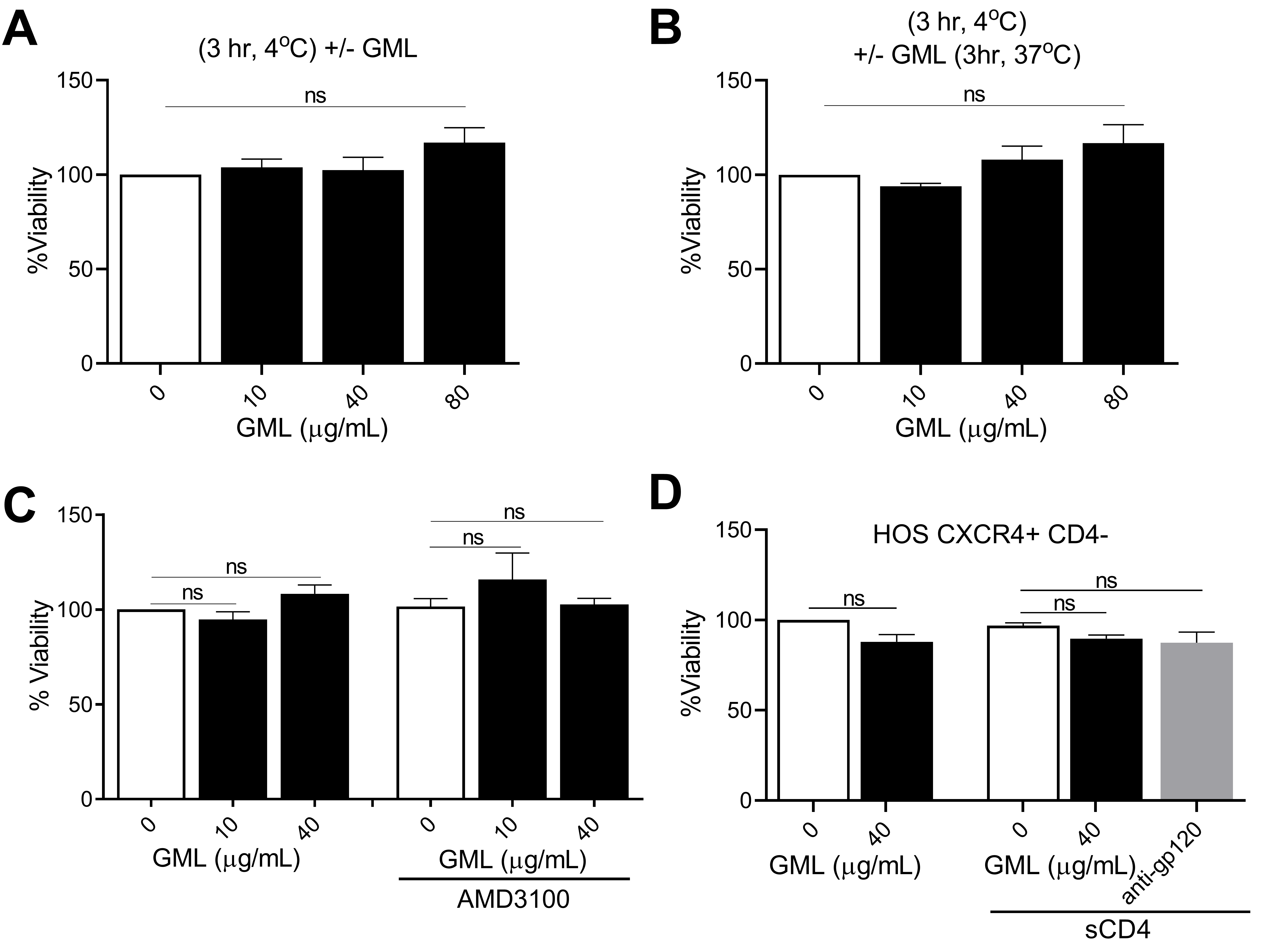

Supplement: FIG S2 [file mBio.00686-20-sf002.tif]

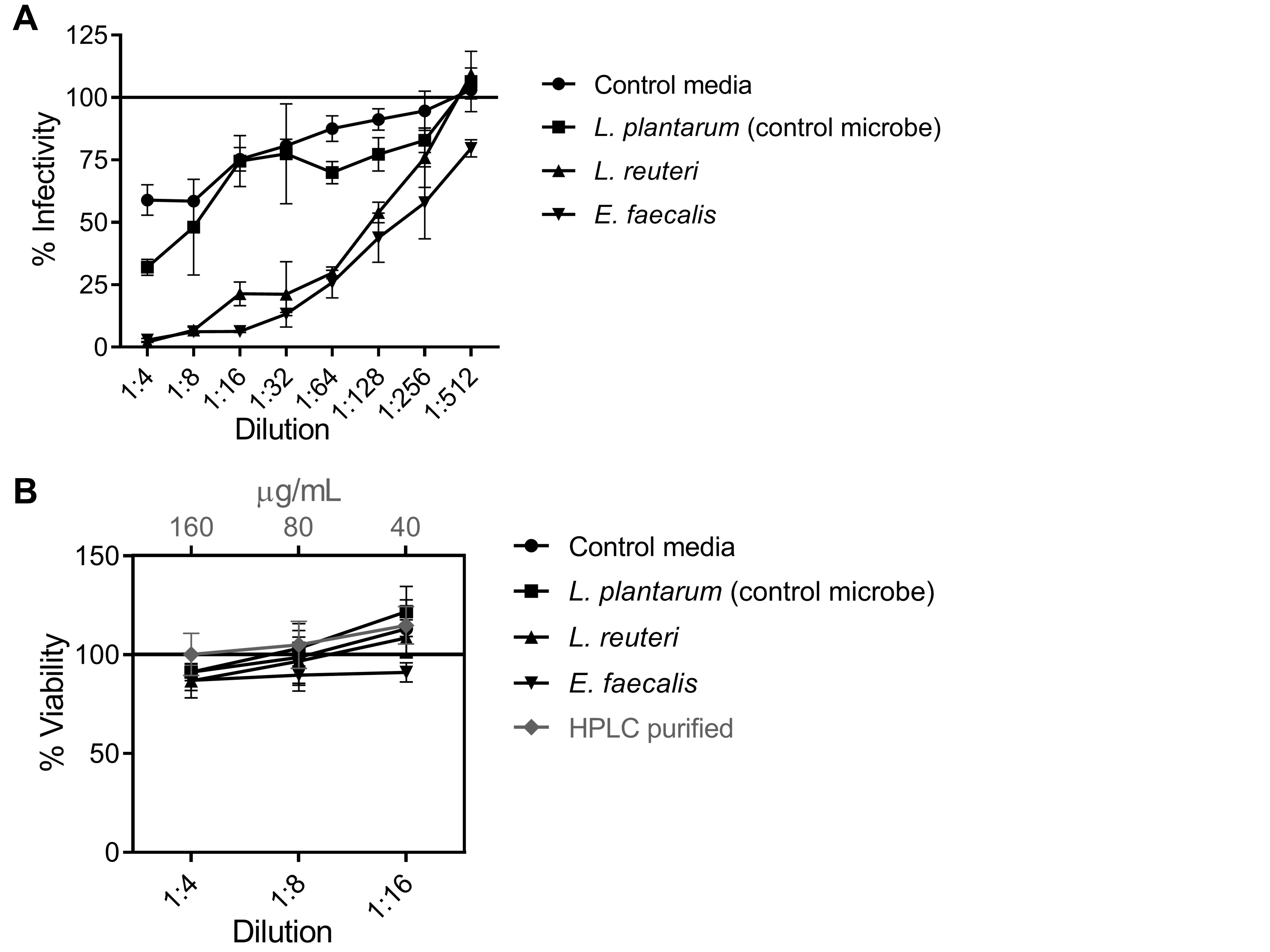

Supplement: FIG S3 [file mBio.00686-20-sf003.tif]
